# Short-Term Efficacy in Polypoidal Choroidal Vasculopathy Patients Treated With Intravitreal Aflibercept or Conbercept

**DOI:** 10.3389/fmed.2022.835255

**Published:** 2022-02-17

**Authors:** Yin Xue, Cai Qinhua

**Affiliations:** Department of Ophthalmology, The First Affiliated Hospital of Soochow University, Suzhou, China

**Keywords:** aflibercept, conbercept, polypoidal choroidal vasculopathy, best-corrected visual acuity, polyp regression

## Abstract

**Purpose:**

To compare the short-term efficacy in patients with polypoidal choroidal vasculopathy (PCV) treated using either aflibercept or conbercept.

**Methods:**

This prospective study included 41 patients with treatment-naive PCV (42 eyes). All the patients were treated with either aflibercept or conbercept using an initial series of 3 monthly loading injections. Changes in the best-corrected logMAR visual acuity (BCVA) and anatomic outcomes were evaluated at 3 months.

**Results:**

BCVA was improved with reduction in central choroidal thickness (CCT), central foveal thickness (CFT), and subretinal fluid (SRF) after 3 monthly loading injections in both aflibercept (IVA) and conbercept (IVC) groups. There was no significant difference in either visual or anatomic outcomes between the two groups after 3 months of treatment. However, compared with the IVC group, significantly higher BCVA improvement was observed in the patients in the IVA group with baseline BCVA better than 1. A visual outcome improved ≥3 lines in 13 patients in the IVA group (59%), and 9 patients in the IVC group (45%). A relatively high proportion of polyp regression was observed in the IVA group (63%) compared with the IVC group (55%) *via* OCTA.

**Conclusions:**

Visual and anatomic outcomes were significantly improved in both IVA and IVC groups, but the results suggest a potentially superior short-term response in the IVA group.

## Introduction

Polypoidal choroidal vasculopathy (PCV) is a subtype of neovascular age-related macular degeneration (nAMD). It differs from typical choroidal neovascularization (CNV) and is defined by a branching vascular network (BVN), polyps, hemorrhagic pigment epithelial detachment (PED), and sub-retinal fluid (SRF) (1, 2).

The prevalence of PCV is higher in Asian people than in Caucasians (3), and the disease course varies between races. In China, the majority of PCV cases were unilateral, with macular polyps and occurred in males, suggesting a less favorable course than in white people (4).

In the past decade, photodynamic therapy (PDT) with verteporfin and intravitreal injection of anti-vascular endothelial growth factor (VEGF) has been widely recommended to treat PCV (5, 6). Availability and long-term side effects of PDT have led to anti-VEGF agents being considered as the first line of therapy (7), but optimal treatment for PCV remains unclear.

In China, the Food and Drug Administration-approved anti-VEGF agents are aflibercept, conbercept, and ranibizumab. Ranibizumab is a humanized, affinity-matured monoclonal antibody, with an antigen-binding fragment (Fab) against all isoforms of VEGF-A, but with weak efficacy in the regression of polyps (8). In contrast toranibizumab, aflibercept, and conbercept are recombinant fusion proteins. As promising new treatments for PCV, it is important to evaluate the clinical efficacy of aflibercept and conbercept (5, 9). Optimal treatment for PCV requires further analysis, particularly with different types of an anti-VEGF agent. This study was conducted to compare short-term changes in eyes treated using aflibercept with those in eyes treated using conbercept.

## Methods

### Study Design

This study enrolled patients with PCV who visited the First Affiliated Hospital of Soochow University between October 2020 and February 2021. The study was approved by the Institutional Review Board of The First Affiliated Hospital of Soochow University and abided by the tenets of the Declaration of Helsinki. The study was registered in the Chinese Clinical Trial Registry as ChiCTR2100048700. This was a prospective study. The enrolled patients and the doctor who collected and analyzed data were double-blinded. The patients with PCV were randomly assigned to receive either aflibercept or conbercept in a one-to-one ratio.

### Patients

**Figure 1:**
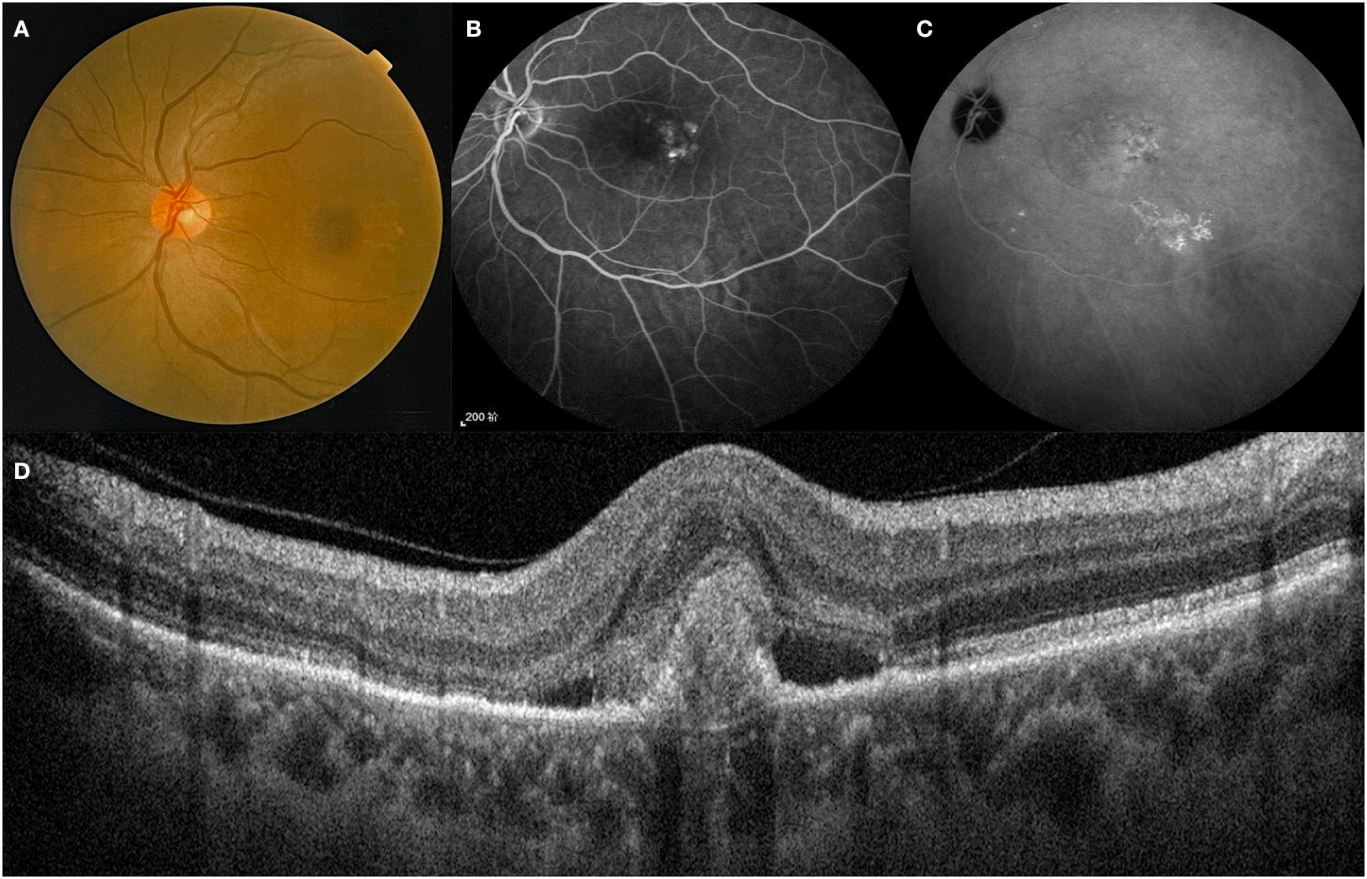
A treatment-naïve patient with PCV. Lesion of the posterior pole on fundus color photography **(A)**. Polyps and BVN on FFA and ICGA **(B,C)**. B-Scan showing the structures of a polyp between RPE and Bruch's membrane **(D)**. BVN, branch vascular network; FFA, fundus fluorescein angiography; ICGA, indocyanine green angiography.

The patients were diagnosed with PCV based on clinical manifestations combined with optical coherence tomography (OCT) and indocyanine green angiography (ICGA) (Figure 1). The patients with treatment-naïve PCV were included in this study. The exclusion criteria were (1) diagnosis of other retinal disease (2) presence of severe cataract, vitreous hemorrhage, or other media opacity (3) previous history of intravitreal injection or PDT (4) internal eye surgery within the previous 3 months (5) history of recent thromboembolic events (6) <18 years of age or inability to act independently.

### FFA/ICGA

FFA/ICGA (HRA-II; Heidelberg Engineering, Dossenheim, Germany) was conducted after intravenous injection of 5-ml sodium fluorescein (Akorn, Copiague, NY, USA) and 5-ml indocyanine green (Ruidu, Dan Dong, Liao Ning, China). During the first minute, the photographs were taken continuously, and then every 1 min in a 5-min interval of FFA/ICGA, and then at 5–10-min intervals in the remaining time, which was 15 min of FFA and 30 min of ICGA.

### Treatment and Follow-Up Protocols

Best corrected logMAR visual acuity (BCVA) measurement, fundus photography, fundus fluorescein angiography (FFA), including ICGA, OCT, and OCTA (Optovue, Fremont, CA, USA) were conducted. The patients received 3 loading intravitreal injections at 1-month intervals of either aflibercept (2 mg/0.05 ml of Eylea^®^ Regeneron, Tarrytown, NY, USA) or conbercept (0.5 mg/0.05 ml of Lumitin, Chengdu Kanghong Biotech Co., Ltd, China). All the patients were followed up for 3 months on a monthly basis after the initial treatment. The patients who were intravitreally injected with aflibercept were defined as the IVA group and those who were intravitreally injected with conbercept were defined as the IVC group. Follow-up examinations, including BCVA, fundus photography, OCT, and OCTA, were performed 1, 2, and 3 months after the first injection.

### Outcome Measures

The primary outcome was BCVA improvement after 3 monthly loading intravitreal injection. The secondary outcome was changes in anatomic structure. OCT was performed to determine the anatomic structure, including central choroidal thickness (CCT), central foveal thickness (CFT), and subretinal fluid (SRF) during the follow-up period. Proportion of polyp regression was detected using OCTA at 3 months after initial IV. The inner boundary of the choroid was set as the hyperreflective line of Bruch's membrane, while the outer boundary was set as the chorioscleral interface. The vertical distance between the inner and outer borders beneath the fovea was defined as CCT. CFT was measured between inner limiting membrane and RPE. The maximum thickness subretinal fluid was defined as SRF. The values were provided from the report or measured with the caliper tool of OCTA.

### Statistical Analyses

Statistical analysis was performed using SPSS software version 13.0 (SPSS Inc., Chicago, Illinois, USA). These small sample data have been tested by normal distribution. In normal distribution data, differences between groups were analyzed using a two-tailed unpaired *t*-test or ANOVA. Multiple comparisons were made using a *post-hoc* test. Non-normal data were analyzed with non-parametric test. A value of *p* < 0.05 was considered statistically significant.

## Results

### Baseline Characteristics

During the study period, a total of 58 eyes of 56 patients diagnosed with PCV were included in this study at the First Affiliated Hospital of Soochow University. Due to attrition, 42 eyes of 41 patients remained in the study after 3 months. Of these, 22 eyes were included in the IVA group and 20 eyes in the IVC group.

Table 1 compares the baseline characteristics between the IVA and IVC groups. The age, gender, prevalence of diabetes or hypertension, BCVA, CCT, CFT, and SRF were not significantly different between the two groups.

**Table 1:** Basic characteristics of PCV patients.

	**IVA**	**IVC**	***p***
Age	66.86 ± 7.55	63.25 ± 8.09	0.142
Gender			
Male/Female	13/9	13/7	0.473
BCVA (LogMAR)	0.93 ± 0.11	1.03 ± 0.12	0.545
CCT (μm)	308.4 ± 63.54	294.9 ± 58.75	0.878
CFT (μm)	317.4 ± 144.2	320.3 ± 137.7	0.989
SRF (μm)	214.5 ± 137.1	209.4 ± 109.6	0.977
Diabetes	4 (18%)	4 (21%)	0.884
Hypertension	8 (36%)	6 (32%)	0.671

*BCVA, best corrected visual acuity; CCT, central choroidal thickness; CFT, central foveal thickness; SRF, sububfoveal retinal fluid*.

### Visual Outcome

**Figure 2:**
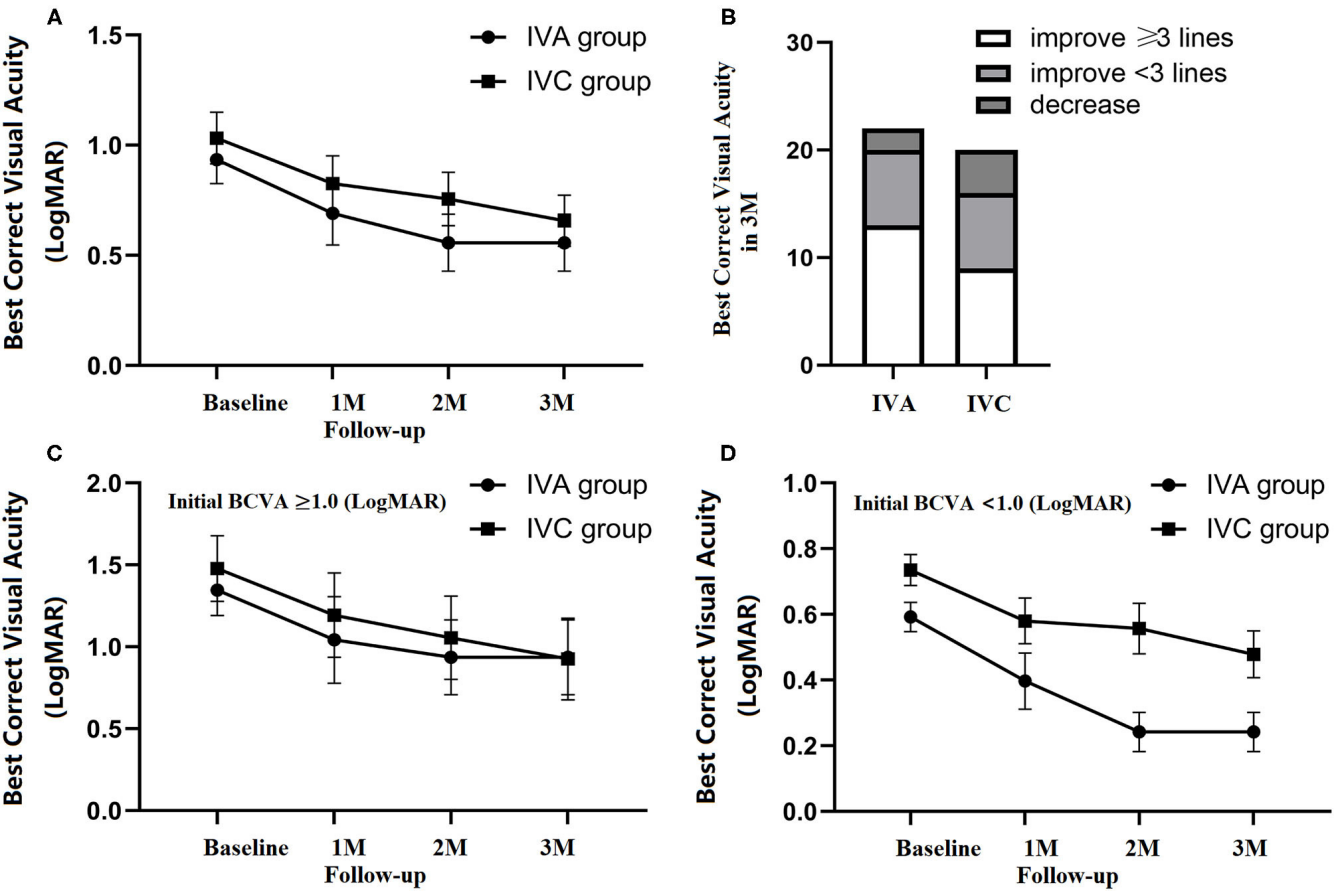
Changes in visual outcomes throughout the 3-month follow-up period. An improvement in BCVA (logMAR) in the IVA (22 eyes) and IVC groups (20 eyes) at 1, 2, and 3 three monthly (M) loading intravitreal injections with no significant difference between the two groups **(A)**. Final BCVA was classified as increased of ≥3 lines, <3 lines and decreased at 3 months **(B)**. Baseline BCVA was classified into ≥1 and <1 subgroups. No significant difference was observed between IVA and IVC in the ≥1 group **(C)**. In the <1. subgroup, final BCVA was significantly better in IVA than IVC (unpaired *t*-test, *p* = 0.014) **(D)**. All data are expressed as means and standard errors of the means (SEM).

From the baseline to 3 months after initial injection, BCVA improved from.93 ± 0.11 to.55 ± 0.13 in the IVA group, and from 1.03 ± 0.12 to.66 ± 0.12 in the IVC group (Figure 2A). There was no significant difference in the visual outcome between the two groups at 3 months.

Compared with initial BCVA, the patients were classified according to their improvement in BCVA at 3-month follow-up, including improvements of ≥3 lines, <3 lines, and decreased acuity. Three months after the initial injection, BCVA improved ≥3 lines in 13 patients in the IVA group (59%), and 9 patients in the IVC group (45%) (Figure 2B).

Of all the patients, two differentiated the subgroup of baseline BCVA (≥1 and <1). In the ≥1 subgroup, BCVA was 1.35 ± 0.15 (baseline), 1.04 ± 0.26 (1M), 0.94 ± 0.23 (2 M), and 0.94 ± 0.22 (3 M) in the IVA group, while 1.48 ± 0.20 (baseline), 1.19 ± 0.26 (1 M), 1.06 ± 0.25 (2 M), and 0.93 ± 0.25 (3 M) in the IVC group. In the <1. subgroup, BCVA was 0.59 ± 0.04 (baseline), 0.40 ± 0.0914 (1 M), 0.24 ± 0.06 (2 M), and 0.24 ± 0.06 (3 M) in the IVA group, while BCVA was 0.74 ± 0.05 (baseline), 0.58 ± 0.07 (1 M), 0.56 ± 0.08 (2 M), and 0.48 ± 0.07 (3 M) in the IVC group (Figures 2C,D). Significant visual improvement was observed in the patients with baseline BCVA better than 1, in both IVA and IVC groups (*p* = 0.001 and *p* = 0.026, respectively), and the improvement was significantly higher in IVA than IVC after 3 monthly loading injections (*p* = 0.018) (Figure 2D).

### Polyp Regression

**Figure 3:**
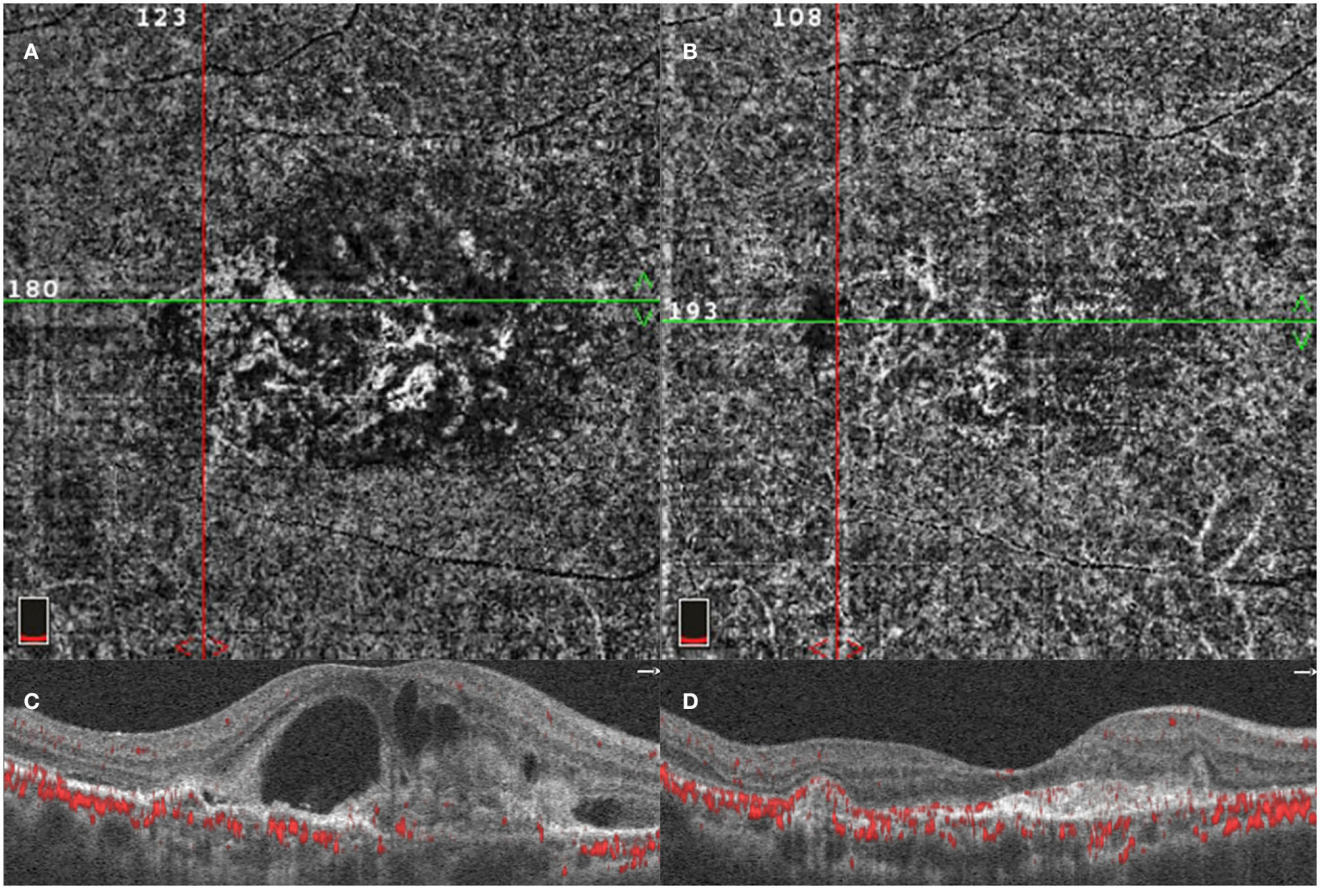
Optical coherence tomography angiography (OCTA) image of PCV lesion. A patient diagnosed with PCV was detected using OCTA before initial injection. OCTA 6-x-6-mm *en face* flow image using a section from the retinal pigment epithelium (RPE) to Bruch's membrane showing the entire BVN, intraretinal fluid, and PCV lesion **(A,C)**. OCTA image of the same lesion after three monthly loading anti-VEGF agent intravitreal injections **(B,D)**.

**Figure 4:**
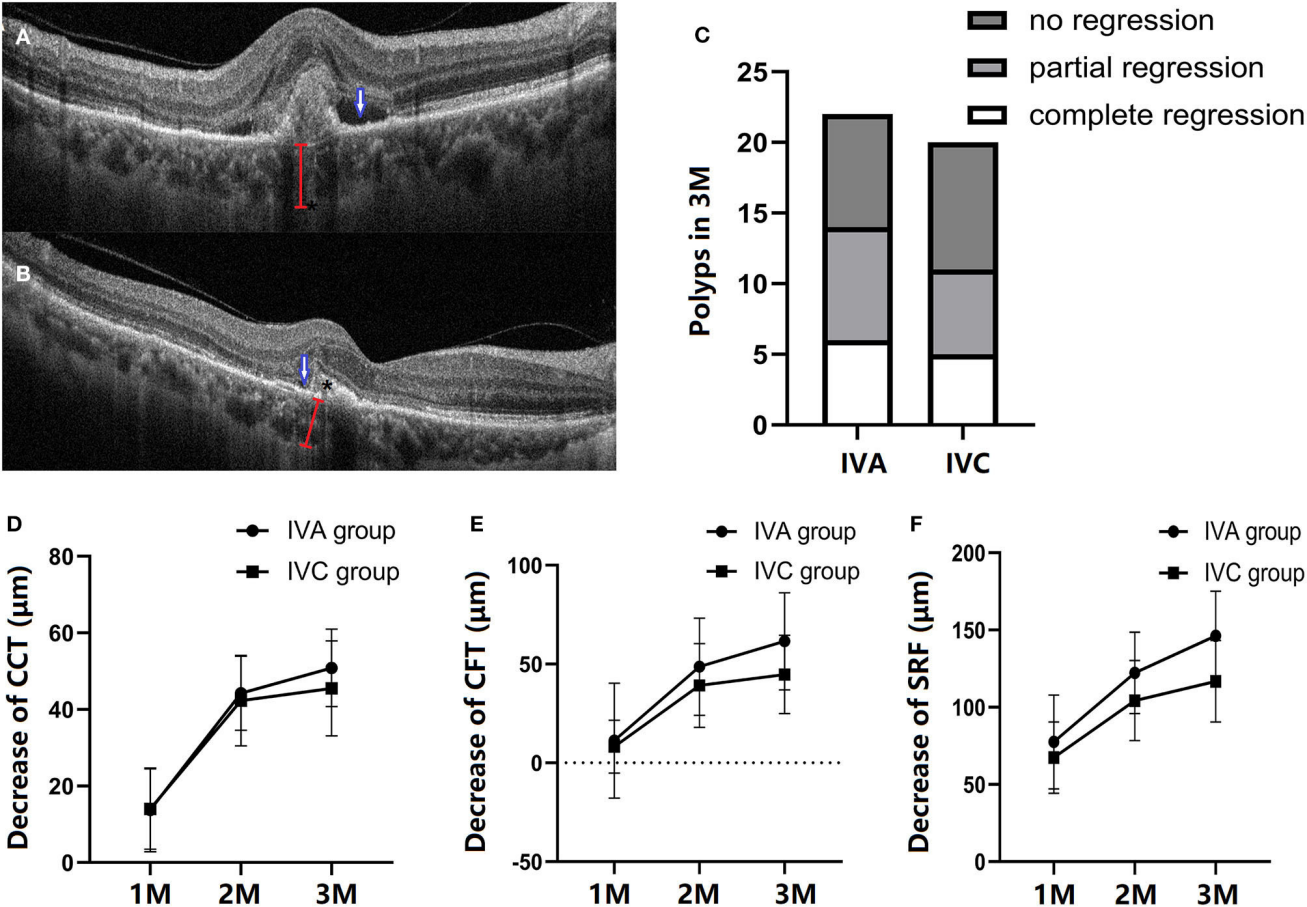
Comparison of structural changes after three monthly (M) loading anti-VEGF agent injections. A representation of patients with PCV in the follow-up period. The outline of polyp (*), subretinal fluid (blue arrow), and choroidal thickness (red line) at the baseline **(A)** and at 3 months (M) **(B)**. At 3 months, polyp regression in the IVA and IVC groups, respectively **(C)**. B-Scan data showing the structure of a polyp between RPE and Bruch's membrane. The decrease of central choroidal thickness (CCT) **(D)**, central foveal thickness (CFT) **(E)**, and subretinal fluid (SRF) **(F)** at 1-M, 2-M and 3-M injections in the follow-up period, respectively. CCT, CFT, and SRF are all decreased after IVA or IVC treatment, with no significant difference between the two groups (one-way ANOVA and unpaired Student's *T*-test).

BVN and polyp lesion were confirmed by ICGA before initial injection in all subjects (Figure 3) and OCT/OCTA was performed at each follow-up time point. At the baseline, polyps were observed in 18 eyes in each of the two groups (Figures 4A,B). After treatment for 3 months, *via* OCT and OCTA, polyp regression was found in the IVA group (63%) and the IVC group (55%). Complete polyp regression was observed in six eyes (27%) in the IVA group, and five eyes (25%) in the IVC group (Figure 4C).

### Retina and Choroid Changes After Treatment

Figure 4A shows retinal and choroidal structure changes after initial injection. Compared with baseline measurements, the CCT, CFT, and SRF were significantly decreased at 3 months in both IVA and IVC groups. CCT was decreased by 50.86 ± 10.10 and 45.50 ± 12.28 μm in the IVA and IVC groups, respectively (Figure 4D). The CFT decreased by 61.50 ± 24.56 and 44.70 ± 19.81 μm in IVA and IVC groups, respectively (Figure 4E), and SRF was significantly reduced by 146.3 ± 28.86 and 116.8 ± 26.46 μm in IVA and IVC groups, respectively (Figure 4F). However, these differences between the two groups were not significant.

### Complication

No severe complications were observed during the follow-up period, such as stroke, endophthalmitis, retinal detachment, and vitreous hemorrhage. But 3 obvious subconjunctival hemorrhage cases were found in both IVA and IVC groups.

## Discussion

PCV occurs in 22–62% of Asians (10). In the PLANET study, IAI monotherapy, with 3 monthly injections of 2-mg IAI, is of functional and anatomical benefits in eyes with PCV (11). Conbercept monotherapy is also effective in the treatment of PCV (12). Optimal treatment for PCV requires further analysis, particularly with different types of the anti-VEGF agent.

Aflibercept and conbercept are common anti-VEGF agents, which are approved by the Food and Drug Administration in China, but their efficacy in the treatment of PCV has not been reported in a comparative study to date. In this study, we have demonstrated that aflibercept and conbercept are both effective in improving function and structure of the visual system in PCV, especially in patients with relatively high baseline BCVA (better than logMAR 1.0). Furthermore, we found no significant difference between these interventions in terms of BCVA after three monthly loading injections. At each of the three 1-month follow-up periods, retinal and choroidal structure changes were similar in the two groups.

Although there was no statistically significant difference in outcomes between IVA and IVC groups, the results suggest that intravitreal aflibercept injection is superior to intravitreal conbercept injection. In addition to the IVA advantage in patients with better baseline BCVA (outlined above), BCVA improvement was at least three lines greater in the IVA than the IVC group. In addition, a relatively high proportion of patients with polyp regression was found in the IVA group compared with the IVC group. Qu et al. found a significantly higher rate of polyp regression with conbercept at 12 months (13), suggesting that the IVA advantage may apply in the short but not longer term.

In addition to VEGF, underlying inflammation is a possible pathogenic mechanism in PCV, since patients with this condition have a higher plasma level of inflammatory chemokines (14). The various outcomes of PCV depend on a range of etiologies (15). As a structurally similar anti-VEGF to conbercept, aflibercept is a soluble decoy receptor fusion protein, which not only binds to VEGF-A, VEGF-B, but also to placental growth factor (PlGF) with higher affinity (16). Chen et al. reported that PlGF is a susceptibility gene for nAMD but not PCV in a Chinese population (17). Nevertheless, short-term aflibercept injection therapy not only leads to VEGF and PlGF suppression but results in reduction of IL-6 and platelet-derived growth factor (18).

Traditionally, the relatively thin ischemic choroid is thought to lead to VEGF upregulation. Recently, PCV has been implicated in “pachychoroidopathy,” distinct from typical nAMD (19, 20). Nakashizuka et al. reported a lack of VEGF positivity in the vascular endothelial cells of PCV (21), and PCV treatment with repeated intravitreal anti-VEGF agent is less effective and has a less favorable anatomical response than in the treatment of typical nAMD (22). In addition to anti-VEGF, suppression of P1GF and other inflammatory factors might enhance the efficacy of aflibercept in treating PCV.

To the best of our knowledge, in angiography, PCV has been classified into polypoidal CNV (Type 1 PCV) and idiopathic PCV (Type 2 PCV), and, in early treatment stages, these two forms respond differently to anti-VEGF treatment. Type 1 PCV was characterized by multiple polypoidal dilatations at the termini of prominent BVNs, while Type 2 PCV showed weak BVN and fewer polyps (23). A higher percentage of polyp regression is a more effective response to aflibercept in polypoidal CNV than idiopathic PCV (24). In this study, one major limitation is that no further analysis was performed within PCV subtypes, such as location and type. Future research with a bigger sample size study and subgroup analysis should be conducted to confirm the present results.

The other limitation is that polyps regression was assessed *via* OCTA. Although Kim reported that OCTA might be better than ICGA in correctly identifying polypoidal lesions (25), it would be more accurate and reliable with ICGA. Unfortunately, most patients refused ICGA after 3 injections.

In addition, the high-dose anti-VEGF agent yields more significant functional and anatomical improvements, especially in short-term follow-up (13, 26). In this study, the two anti-VEGF agents, aflibercept (2 mg/0.05 ml) and conbercept (0.5 mg/0.05 ml) were dissolved in different concentrations. We assumed that the patients in the high-dose group received more doses of anti-VEGF agent. All these factors may have contributed to a superior short-term response in the IVA group.

## Conclusion

In conclusion, visual and anatomic outcomes were significantly improved in both IVA and IVC groups after three monthly loading injections. Anti-VEGF therapy involving either aflibercept or conbercept results in visual gain with a reduction in CCT, CFT, and SRF in the patients with PCV. However, our results suggest that visual and anatomical gain may be better in intravitreal aflibercept compared with conbercept injection in some cases. In the patients with better baseline BCVA, aflibercept leads to a more significant visual improvement. In addition, of the proportion of the patients with visual improvement ≥3 lines and with polyp regression were relatively high in the IVA group. These results suggest a superior short-term response to aflibercept treatment for PCV.

## Data Availability Statement

The original contributions presented in the study are included in the article/supplementary material, further inquiries can be directed to the corresponding author.

## Ethics Statement

The studies involving human participants were reviewed and approved by Institutional Review Board of the First Affiliated Hospital of Soochow University. The patients/participants provided their written informed consent to participate in this study.

## Author Contributions

YX contributed to data acquisition, analysis, and manuscript writing. CQ contributed to design, data analysis, and manuscript writing. Both authors contributed to the article and approved the submitted version.

## Funding

This study was supported by the National Natural Science Foundation in China (Grant No. 82101120), the Jiangsu Provincial Natural Science Foundation (Grant No. BK20210095), and the Soochow Livelihood Technology Project (Grant No. SYS2020114).

## Conflict of Interest

The authors declare that the research was conducted in the absence of any commercial or financial relationships that could be construed as a potential conflict of interest.

## Publisher's Note

All claims expressed in this article are solely those of the authors and do not necessarily represent those of their affiliated organizations, or those of the publisher, the editors and the reviewers. Any product that may be evaluated in this article, or claim that may be made by its manufacturer, is not guaranteed or endorsed by the publisher.
